# Limits of patient isolation measures to control extended-spectrum beta-lactamase–producing Enterobacteriaceae: model-based analysis of clinical data in a pediatric ward

**DOI:** 10.1186/1471-2334-13-187

**Published:** 2013-04-24

**Authors:** Matthieu Domenech de Cellès, Jean-Ralph Zahar, Véronique Abadie, Didier Guillemot

**Affiliations:** 1 Unité de Pharmacoépidémiologie et Maladies Infectieuses, Institut Pasteur, Paris, France; 2 U657, Inserm, Paris, France; 3 Paris 6: Univ. Pierre et Marie Curie, Cellule Pasteur UPMC, Paris, France; 4 Unité d’hygiène hospitalière, Service de microbiologie, CHU Necker-Enfants Malades, Paris, France; 5 Université Paris Descartes, Paris, France; 6 Service de Pédiatrie générale, CHU Necker-Enfants Malades, Paris, France; 7 EA 4499, Université de Versailles–Saint-Quentin-en-Yvelines, Versailles, France; 8 Unité Fonctionnelle de Santé Publique, Hôpital Raymond-Poincaré, Assistance Publique–Hôpitaux de Paris, Garches, France

**Keywords:** ESBL-E, Healthcare epidemiology, Bacterial pathogens, Mathematical modeling, Statistical inference

## Abstract

**Background:**

Extended-spectrum beta-lactamase–producing Enterobacteriaceae (ESBL-E) are a growing concern in hospitals and the community. How to control the nosocomial ESBL-E transmission is a matter of debate. Contact isolation of patients has been recommended but evidence supporting it in non-outbreak settings has been inconclusive.

**Methods:**

We used stochastic transmission models to analyze retrospective observational data from a two-phase intervention in a pediatric ward, successively implementing single-room isolation and patient cohorting in an isolation ward, combined with active ESBL-E screening.

**Results:**

For both periods, model estimates suggested reduced transmission from isolated/cohorted patients. However, most of the incidence originated from sporadic sources (i.e. independent of cross-transmission), unaffected by the isolation measures. When sporadic sources are high, our model predicted that even substantial efforts to prevent transmission from carriers would have limited impact on ESBL-E rates.

**Conclusions:**

Our results provide evidence that, considering the importance of sporadic acquisition, e.g. endogenous selection of resistant strains following antibiotic treatment, contact-isolation measures alone might not suffice to control ESBL-E. They also support the view that estimating cross-transmission extent is key to predicting the relative success of contact-isolation measures. Mathematical models could prove useful for those estimations and guide decisions concerning the most effective control strategy.

## Background

Multidrug-resistant bacteria are a continuing threat in hospital settings, causing a high morbidity and mortality worldwide [1]. Among Gram-negative bacteria, resistance to beta-lactams mainly results from extended-spectrum beta-lactamase, a major group of plasmid-mediated enzymes conferring resistance to the penicillins and first- to third-generation cephalosporins [2]. Extended-spectrum beta-lactamase–producing Enterobacteriaceae (ESBL-E) are becoming increasingly prevalent in hospitals, with consequences now documented in terms of increased mortality and delayed onset of effective therapy [3]. Moreover, it is now recognized that ESBL-E also spread in the community, which can serve as a reservoir for hospitals [4,5]. Strategies to control the spread of multiresistant bacteria, particularly ESBL-E, in hospitals are being debated [6,7], and disparities in infection-control practices have been reported as a consequence [8]. Enhanced barrier precautions are advocated and some authors (albeit not all, see [9]) have reported successful curtailment of outbreaks using those measures [10,11]. In the non-outbreak setting, however, achieving ESBL-E control is more difficult and, to date, the evidence for efficacy of barrier precautions has been scarce [12], and sometimes inconclusive [13].

ESBL-E colonization can result from patient-to-patient transmission and/or emergence or selection during antibiotic therapy. Given the relative importance of these two routes, different interventions can be implemented to control one or both sources [14]. Contact isolation of carriers is typically used to interrupt transmission from detected colonized/infected patients, but is not expected to affect endogenous selection [7]. Contact isolation of patients is currently recommended by several guidelines but concerns exist that isolation might, in some cases, lower the quality of care [15]. Contact isolation can include a wide spectrum of interventions, ranging from barrier nursing (e.g. gowns and gloves) to a full isolation ward with designated staff (i.e. nurse cohorting). It is likely that the impact of any such intervention will depend on locally variable factors (e.g. the case-mix of patients, physical environment, available resources) and the epidemiology of the pathogen [7]. Considering the substantial resources involved in a screening-and-isolation program [13,16], it is of paramount importance to determine their efficacy accurately. In addition, while major efforts have been devoted to understand the impact of interventions on controlling the spread of Gram-positive pathogens, reliable evidence for Gram-negative bacteria, including ESBL-E, is lacking [14].

Herein, we used observational data from a two-phase intervention study in a pediatric ward, during which we successively implemented two contact-isolation strategies (single-room isolation and isolation ward with nurse cohorting), combined with active surveillance for ESBL-E carriage. Using mechanistic modeling, our aims were to gain insight into ESBL-E epidemiology, clarify the role of contact isolation in preventing ESBL-E spread and predict effective interventions in various settings.

## Methods

### Ethics statement

This study used observational data collected as part of systematic routine surveillance procedures in a university hospital ward, with an endemic level of multidrug-resistant bacteria. This surveillance protocol followed the official recommendations of the French Ministry of Health and the French Society for Hygiene (http://sante.gouv.fr/les-infections-nosocomiales-recommandations-aux-etablissements-de-soins.html) and was approved by the Nosocomial Infections Fighting Committee. All the patients’ parents received general information about the hospital infection control strategy at admission. No more information than those collected by routine procedures was used; in particular, no additional individual data, biological collection or sample was required. Therefore, an ethics committee approval was not required for this study.

### Setting and description of interventions

Necker Enfants–Malades is a 650-bed tertiary-care teaching hospital that handles 55 000 admissions per year. The pediatrics department includes a 21-bed unit that admits 300–350 children annually. Approximately half of the children are referred from other hospitals, 20% from other units in the hospital, and the remaining 30% from the emergency department. In May 2009, a whole-ward screening revealed an unusually high ESBL-E prevalence among patients, which prompted the subsequent interventions, beginning in June 2009 and described below. All children admitted from 1 June 2009 through 15 July 2010 were included in our study. All episodes of ESBL-E colonization or infection diagnosed during the stay or up to 2 days after discharge from the pediatric ward were included. During the study period, a rectal swab specimen was obtained at admission and once a week throughout each child’s stay. Swabs were plated on a selective chromogenic medium for ESBL screening (chromID ESBL Agar, bioMérieux, Marcy-l’Etoile, France). Enterobacteriaceae were isolated and identified according to the recommendations of the Comité de l’Antibiogramme de la Société Française de Microbiologie. ESBL production was evaluated with the double-disc synergy test and the Etest (AB Biodisk, Solna, Sweden) for ceftazidime and ceftazidime–clavulanate. Patients with ESBL-E isolated within 48 hours following admission from screening samples or from an infected site were considered imported. Other cases were considered acquired.

### Infection control measures

From June 2009 through February 2010 (first period, P_1_), the following baseline infection-control practices were in place in the pediatrics ward. All children with ESBL-E–positivity in clinical or screening specimens were placed in single rooms and in contact isolation, within 24 h following test results. Briefly, these measures consisted of flagging microbiological reports, charts and doors of rooms of ESBL-E–positive patients with a warning symbol; wearing gowns and gloves when caring for these patients; emphasizing hand hygiene before and after patient contact; and notifying ESBL-E carriage when patients were transferred to another unit. Contact isolation was maintained throughout the entire duration of hospitalization. During the second period (P_2_), from February 2010 to July 2010, a cohorting protocol was added to the aforementioned measures. All known ESBL-E carriers were moved and grouped at one end of the pediatrics ward in an 8-bed unit, and cared for by a dedicated nursing team. The isolation policy was similar to P_1_, except that previously known carriers (i.e. patients with an episode of ESBL-E carriage in the past 6 months) were placed in isolation immediately upon admission.

### Mathematical model

**Figure 1:**
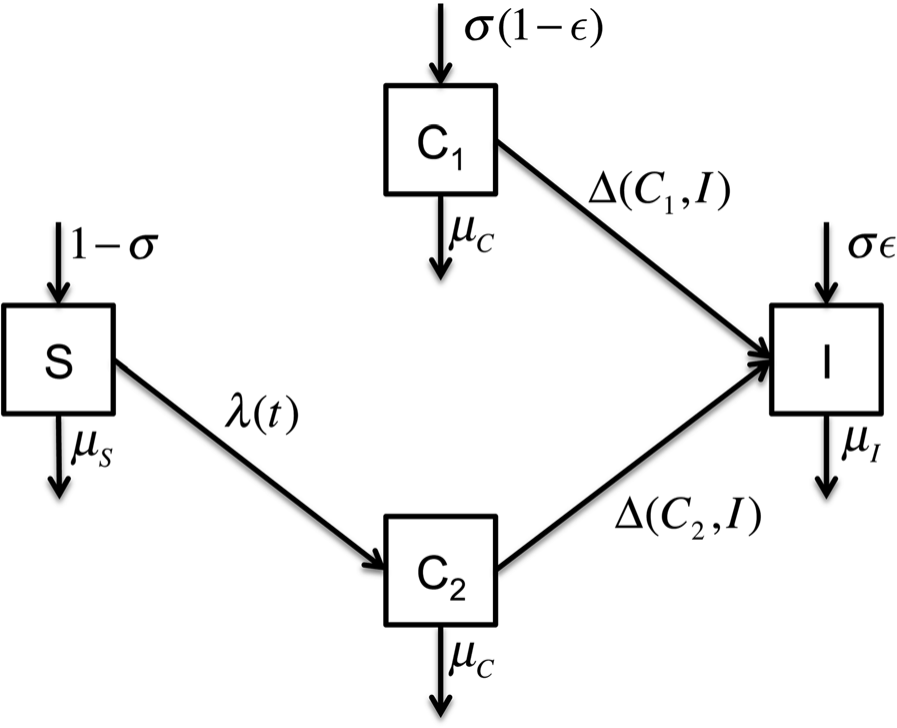
**Model representation.** Compartments represent different epidemiological states. Parameters are defined in Table 1. Arrows indicate transitions between states, which occur at a rate given by the parameter. The term Δ(C_*_,*I)* represents an isolation function, such as Δ(C_*_,I)=δ_*_C_*_ if *I* < N_I_ (at least one isolation bed available), 0 otherwise.

We assessed the effectiveness of isolation measures by means of a stochastic, population-based transmission model, building on previously described models [17,18]. Patients can be in four mutually exclusive states, depending on their colonization and isolation status (Figure 1). Patients can be susceptible (*S*) or colonized, with the latter subdivided as isolated (*I*) or unisolated, distinguished as imported cases (patients colonized at admission *C*_*1*_) or acquired cases (patients acquiring ESBL-E in the unit, *C*_*2*_). Susceptible patients are at risk of acquiring ESBL-E at a rate *λ* = *β*_0_ + *β*_1_(*C*_1_ + *C*_2_) + *β*_2_*I*, where *β*_1_ and *β*_2_ are the transmission rates from unisolated and isolated ESBL-E carriers, respectively, and *β*_0_ represents the non-cross–transmission acquisition rate. Although it is common practice to include both cross-transmission and non-cross–transmission terms in transmission models, their exact interpretation is subtle. In our model, these quantities are just two components of the attack rate: *β*_1_ and *β*_2_ scale that component proportional to the number of carriers (unisolated and isolated, respectively), while *β*_0_ quantifies the component independent of the number of carriers. With respect to ESBL-E dynamics, the cross-transmission characteristic is not whether it is transmitted through the contaminated hands of healthcare workers, environmental contamination, fomites *etc*., but whether its rate depends on previous numbers of carriers in the ward (the so-called colonization pressure [19]). In contrast, sporadic acquisitions are decoupled from the previous ESBL-E dynamics in the ward. Within hospital settings, these acquisitions can include endogenous selection of a previously undetected colonizing strain after antibiotic therapy, which can reasonably be considered to be independent of ESBL-E prevalence in the ward [20,21]. Below, we refer to such acquisitions as sporadic, quantified by the sporadic acquisition rate *β*_0_. Cross-transmission is quantified by the transmission rates *β*_1_ and *β*_2_, and, because isolated ESBL-E–positive patients are managed under special contact precautions, we expect *β*_1_>*β*_2_.

Imported cases are isolated at rate *δ*_1_, and *δ*_2_ for acquired cases, with *δ*_1_>*δ*_2_. When the isolation ward reaches full capacity, the possibility of isolating a patient no longer exists, until an isolated patient is discharged, i.e. the isolation rate is Δ(C_*_,I)=δ_*_C_*_ if *I* < *N*_I_, 0 otherwise. Because the duration of ESBL-E carriage (estimated at 6 months [22]) typically exceeds hospital lengths of stay, we did not account for possible carriage clearance during the stay. The preemptive isolation of previously known carriers in P_2_ is modeled as an additional parameter ***∈***, representing the proportion of colonized inpatients immediately placed in isolation at admission.

### Model parameters

The parameters used in the model are defined in Table 1. Some of them could be computed directly from the dataset. Discharge rates were computed from the patients’ observed lengths of stay recorded in the unit, assuming that all unisolated patients had the same risk of being discharged. Isolation rates were computed as the reciprocal of the mean time to isolation. For imported cases, this time was 2 days during P_1_, and 0 days during P_2_, i.e. we assumed that all colonized inpatients were put in preemptive isolation for this period. For simplicity, we also assumed that all patients put in preemptive isolation during P_2_ were indeed carriers. For acquired cases, because the exact time from acquisition to isolation was unknown, it was assumed to be 4 days. Compared to single-bed isolation rooms, an isolation ward has a clear face validity, meaning that a well-implemented isolation ward with designated staff will effectively prevent all transmission from cohorted patients [23]. However, that assumption does not hold for weaker types of isolation, including single-room isolation, whose effectiveness is *a priori* unclear [18]. Therefore, we set *β*_2_ = 0 for P_2_, but estimated the P_1_ value. Other acquisition parameters, namely *β*_0_ and *β*_1_, and the admission prevalence *σ* were estimated from data for both periods.

**Table 1:** Model parameters for both intervention periods

**Parameter**	**Symbol**	**P**_**1**_	**P**_**2**_
Sporadic acquisition rate	*β*_0_	**0.009 (0.002–0.022)**	**0.008 (0.001–0.015)**
Transmission rate from unisolated patients	*β*_1_	**0.006 (0–0.017)**	**0.006 (0–0.016)**
Transmission rate from isolated patients	*β*_2_	**0.001 (0–0.005)**	0
Admission prevalence	σ	**0.18 (0.14–0.22)**	**0.15 (0.11–0.21)**
Isolation rate for imported and acquired cases	*δ*_1_,*δ*_2_	0.5, 0.25	0.5, 0.25
Fraction of colonized inpatients placed in preemptive isolation	∈	0	1
Discharge rates of susceptible, colonized and isolated patients	*μ*_S_,*μ*_C_,*μ*_I_	0.2, 0.2, 0.13	0.18, 0.18, 0.13
Number of patients	*N*	16	16
Number of isolation beds	*N*_I_	16	8

Parameters in boldface were estimated from data using the iterated filtering algorithm, as explained in the main text. For these parameters, the maximum likelihood estimates (95% confidence intervals) are indicated. Rates are day^–1^.

### Numerical implementation and estimation method

The model was implemented in the pomp package [24], operating in the R environment [25]. Stochastic simulations of the model were performed using Gillespie’s exact algorithm [26]. Parameters were estimated with the iterated filtering algorithm, described elsewhere [27]. Profile likelihoods were used to derive 95% confidence intervals (CI) [28]. Technical details about model implementation and estimation are given in the electronic supplementary material (Additional file 1); the estimation procedure was also verified by using simulated data (Additional file 2).

### Model evaluation

Model fitting was assessed by visual inspection for both weekly point prevalence (i.e. point prevalence on Monday) and weekly incidence data. 10 000 model simulations were used to derive mean values and 95% prediction intervals, and were compared to observed P_1_ and P_2_ data.

## Results

### Parameter estimates and model checking

**Figure 2:**
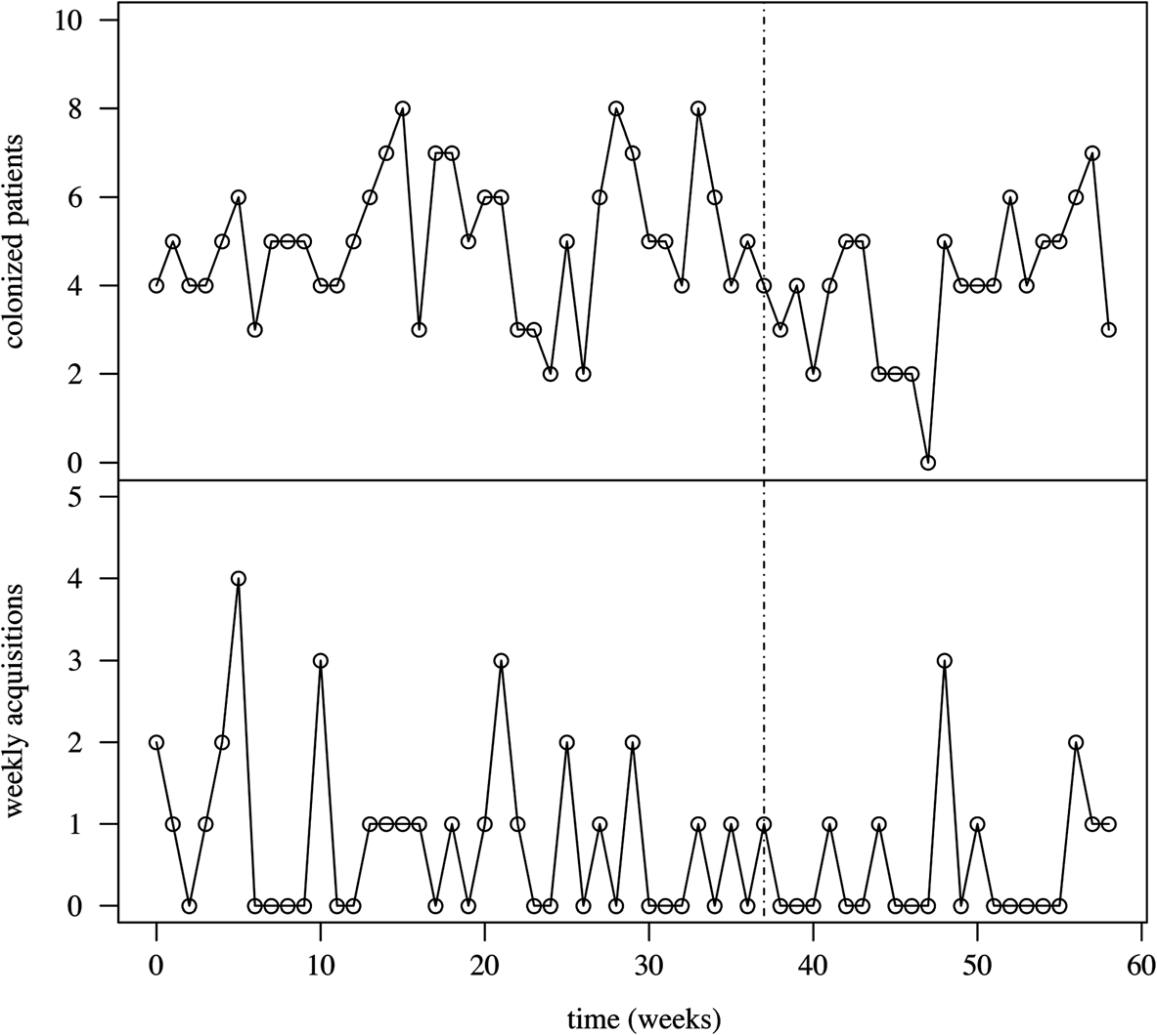
**Time-series for point prevalence and incidence data.** Point-prevalence numbers of colonized patients (upper panel) and cumulated weekly numbers of acquisitions (lower panel) are represented. The vertical dot-dashed line at week 37 indicates isolation-ward implementation.

**Figure 3:**
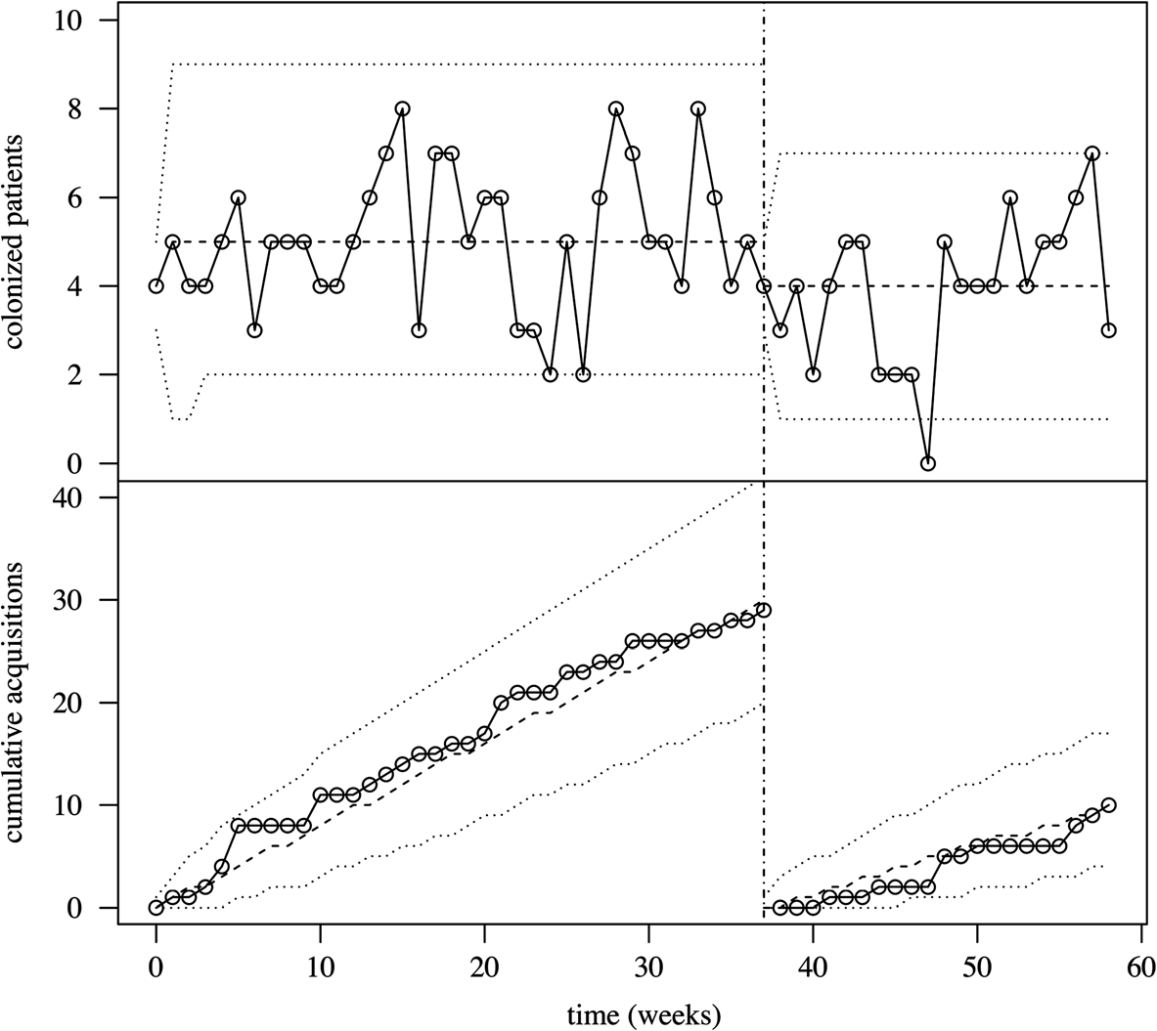
**Model fit to data.** Mean (dashed lines) and 95% prediction intervals (dotted lines) are represented for point-prevalence and weekly incidence data. Point-overlaid continuous lines indicate observed values. The vertical dot-dashed line at week 37 indicates the isolation- ward implementation.

P_1_ and P_2_, respectively, consisted of 4165 and 2305 patient-days (pt-d). The incidence of ESBL-E acquisition was 7.0 per 1 000 pt-d during P_1_ and decreased to 4.3 per 1 000 pt-d during P_2_. A similar trend was observed for ESBL-E prevalence, from 26% during P_1_ to 21% during P_2_ (Table 2). Figure 2 reports the time-series for point prevalence and incidence data. Although estimates for P_1_ were consistent with higher transmission from unisolated patients (*β*_1_ > *β*_2_, Table 1), the difference was not statistically significant compared to isolated patients (95% CI for *β*_1_ − *β*_2_, -0.005–0.018 per day). Moreover, only the sporadic acquisition rate was statistically significant, while transmission rates were not (likelihood ratio tests, null hypotheses *β*_1_ = 0 and *β*_2_ = 0, p-values 0.16 and 0.35, respectively). Admission prevalence was estimated at 0.18. Model assessment suggested very good fit to point prevalence and incidence data (Figure 3). The fitted model predicted a total of 30 (20–42) acquisitions, i.e. a 7.2 (4.8–10.1)/1 000 pt-d incidence, compared with 29 observed acquisitions, i.e. 7.0/1 000 pt-d incidence, corresponding to a predicted 3.4/1 000 pt-d incidence due to sporadic sources, 2.6/1 000 pt-d to transmission from unisolated carriers and 1.2/1 000 pt-d from isolated carriers. Therefore, sporadic acquisitions accounted for nearly 50% of new cases in the ward during P_1_.

**Table 2:** Epidemiological data for both intervention periods

**Data**	**P**_**1**_	**P**_**2**_
Screening policy	all patients, at admission and every Monday	all patients, at admission and every Monday
Isolation policy	within 24 h following test positivity	preemptive isolation of colonized inpatients, otherwise within 24 h following positivity
Type of isolation	single room	8-bed ward
Overflow policy	—	admissions stopped
Admissions, no.	690	333
Days (pt-d)	260 (4165)	148 (2305)
Mean occupancy (range)	16 (6–22)	16 (9–20)
Mean staff-to-patient ratio (range)	0.89 (0.45–2.2)	0.85 (0.5–1.5)
Mean length of stay (range), days	6 (0–83)	6.7 (0–71)
Acquisitions, no.	29	10
*K. pneumoniae*	20	7
*E. coli*	4	0
Other species	5	3
Incidence, per 1 000 pt-d	7.0	4.3
*K. pneumoniae*	4.8	3.0
*E. coli*	1.0	0.0
Other species	1.2	1.3
Prevalence, %	26	21
*K. pneumoniae*	18	15
*E. coli*	3	2
Other species	5	4

The prevalence was computed as the ratio of the total number of colonized patient-days to the total number of patient-days for each period. The incidence was computed as the ratio of the number of acquisitions to the total number of patient-days for each period. The staff-to-patient ratio was computed as the daily ratio of the number of staff members (nurses and ward assistants) to the number of patients. pt-d: patient-days.

During P_2_, the isolation ward had little impact on acquisition rates: the sporadic acquisition rate remained the only statistically significant source of acquisitions in the ward, while transmission from unisolated patients remained unchanged and insignificant (likelihood ratio test, null hypothesis *β*_1_ = 0, p-value 0.2). Admission prevalence was estimated at 0.15 for this period. Again, model fitting was adequate, with a total of 10 (4–18) predicted acquisitions, a 4.2 (1.7–7.6)/1 000 pt-d incidence, compared to 10 observed acquisitions (Figure 3), corresponding to a predicted 3.4/1 000 pt-d incidence resulting from sporadic sources and 0.8/1 000 pt-d a consequence of transmission from unisolated carriers, while the incidence due to cohorted patients was hypothetically set at 0. Crucially, the incidence resulting from sporadic acquisitions was unaffected by the isolation-ward implementation.

Although we estimated parameters separately for P_1_ and P_2_, estimations of acquisition parameters for the entire period are relevant. Doing so, *β*_1_ was estimated at 0.006 (0–0.015) per day and *β*_0_ at 0.008 (0.002–0.015) per day. Thus, transmission from unisolated carriers remained statistically insignificant but a trend was observed (likelihood ratio test, null hypothesis *β*_1_ = 0, p-value 0.08).

### Comparing interventions

Because the preintervention period was not observed, a baseline incidence could not be computed to compare the relative efficacies of the two interventions. Furthermore, the isolation-policy change for imported cases during P_2_ (preemptive isolation of previously known carriers) might obfuscate the precise contribution of the isolation ward to the observed incidence decline. Therefore, in addition to the observed interventions during P_1_ and P_2_, we used model simulations to predict the impact of two hypothetical situations: no isolation whatsoever, and an isolation ward without preemptive isolation, as summarized in Table 3. In the absence of isolation measures (*β*_2_ = *β*_1_), the predicted incidence was 14.6/1 000 pt-d, a two-fold increase compared to P_1_. Had the isolation ward been implemented without preemptive isolation of known carriers, the predicted incidence was 5.9/1 000 pt-d, 16% lower than the observed incidence during P_1_. Overall, these simulations suggested that single-room isolation effectively lowered ESBL-E incidence and that the isolation ward would have had little benefit compared to single-room isolation, had the isolation policy been similar. However, broadly overlapping prediction intervals, reflecting parameter-estimate uncertainty (particularly the insignificance of transmission parameters), preclude a definitive conclusion regarding interventions from these model simulations.

**Table 3:** Comparing interventions

**Intervention**	**Predicted incidence (per 1 000 pt-d)**	**Observed incidence (per 1 000 pt-d)**
No isolation	14.3 (2.4–30)	—
Single-room isolation	7.2 (2.5–13.9)	7.0
Isolation ward without preemptive isolation	5.9 (2–10.5)	—
Isolation ward with preemptive isolation	4.2 (0.9–7.4)	4.3

Predicted intervals were based on 1000 model simulations. Dashes indicate hypothetical interventions that were only simulated, and, therefore, not observed. pt-d: patient-days.

### Predicting the impact of multifaceted interventions

**Figure 4:**
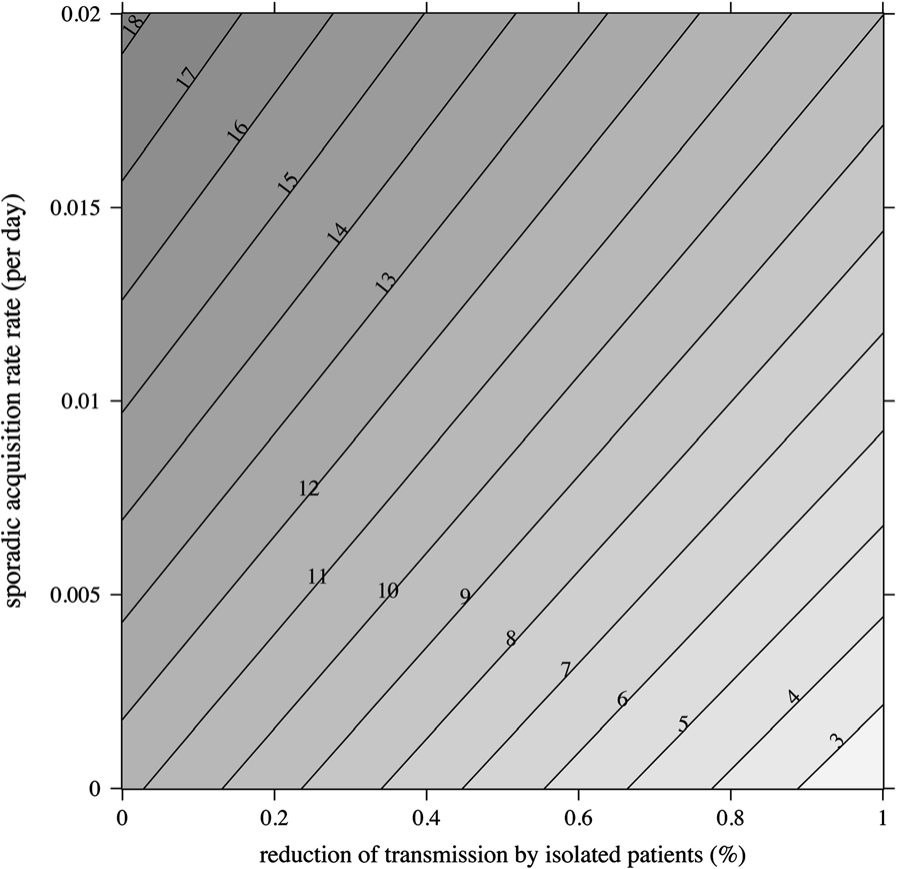
**Predicted impact of multifaceted interventions.** Contour plot of incidence is represented for different levels of isolation effectiveness (1 − *β*_2_/*β*_1_, *x*-axis) and sporadic acquisition rate (*β*_0_, *y*-axis). Increasing incidence values are indicated by ever darker shades of gray, black lines delineate incidence contours with the corresponding threshold value (per 1 000 patient-days). For these simulations, *β*_1_ was set to 0.006 per day, other model parameters were those in P_1_ (Table 1).

We attempted to investigate the expected impact of contact isolation (aimed at reducing patient-to-patient transmission) in combination with measures targeting other acquisition sources (e.g. antimicrobial stewardship program to reduce endogenous acquisition). We used incidence of colonization per 1 000 pt-d as the outcome criterion and simulated the expected impact of contact isolation for various levels of isolation effectiveness (0–100% effective) and different values for sporadic acquisition (Figure 4). When sporadic acquisition sources are high, the model predicted that even substantial efforts to interrupt transmission from isolated patients would have limited impact on lowering the ESBL-E incidence. For example, for *β*_0_ = 0.02 acquisitions per susceptible patient per day, no contact isolation resulted in an 18/1 000 pt-d incidence, whereas completely effective contact isolation yielded a 10/1 000 pt-d incidence, i.e. a 44% incidence reduction. Conversely, when acting simultaneously on sporadic sources of acquisition and cross-transmission, a better control would be expected. Assuming that sporadic sources are fully contained, totally effective contact isolation would be expected to reach a 2/1 000 pt-d incidence, compared to 11/1 000 pt-d without any contact isolation, an 82% decline.

### Impact of screening policy at admission

**Figure 5:**
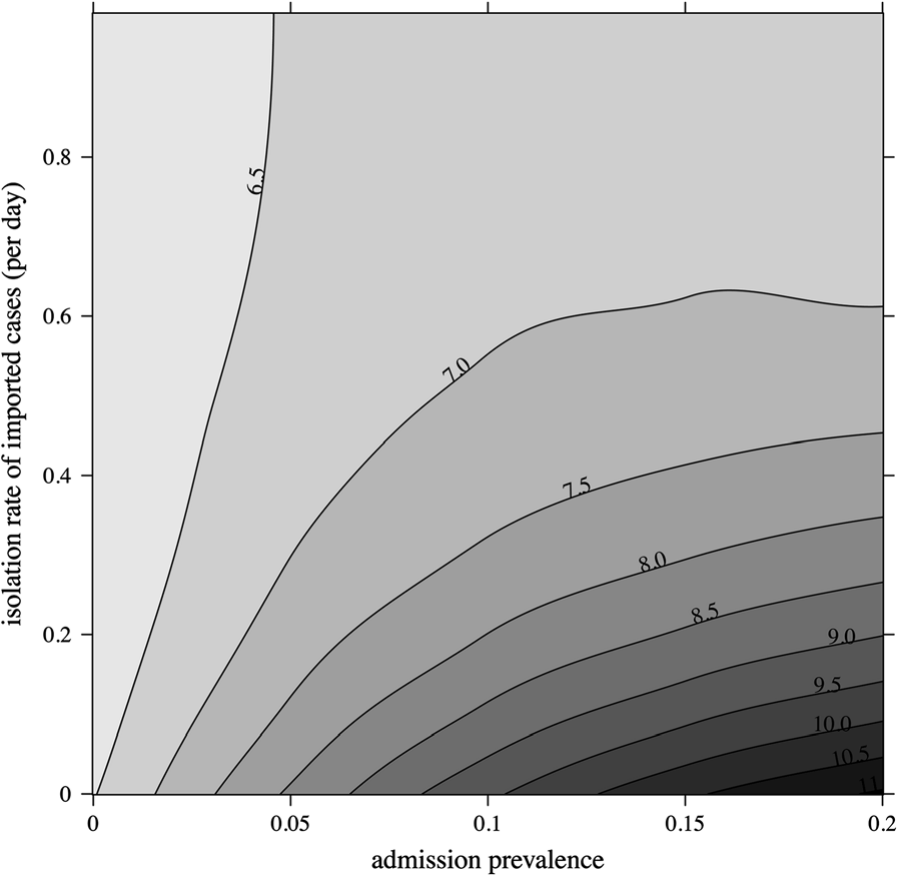
**Impact of screening at admission.** Contour plot of incidence is represented for different levels of admission prevalence (*σ*, *x*-axis) and isolation rate of imported cases (*δ*_1_, *y*-axis). Increasing incidence values are indicated by ever darker shades of gray, black lines delineate incidence contours with the corresponding threshold value (per 1 000 patient-days). For these simulations, model parameters values were those estimated or fixed in P_1_ (Table 1).

We investigated the impact of screening and isolating ESBL-E-positive patients at admission while varying the admission prevalence from 0 to 20%, and assuming that cross-transmission occurs in the ward (Figure 5). When the admission prevalence was low, screening patients at admission had little or no benefit, regardless of the isolation rate. For rising admission prevalences, a higher degree of control could be achieved by screening and isolating colonized inpatients more-and-more rapidly. For example, for an admission prevalence of 20%, the predicted incidence was 11/1 000 pt-d without any screening and could be lowered to 6–7/1 000 pt-d when colonized inpatients were detected and isolated within 2 days.

### Sensitivity analysis

For acquired cases, isolation was assumed to occur 4 days after acquisition. However, because the exact acquisition date was unknown, this value was uncertain. Likewise, the value of direct-isolation proportion during P_2_ was uncertain, because previously unknown carriers colonized at admission could have been missed. Therefore, we conducted sensitivity analyses for these two parameters and found that even large variations of their values had little impact on our estimates (Additional file 1).

## Discussion

We used mechanistic modeling to assess the impact of contact-isolation measures to prevent ESBL-E spread in a pediatrics ward based on clinical data covering a 14-month period. ESBL-E incidence and mean prevalence decreased during the study period, and model estimates suggested that these declines were attributable to reduced transmission from isolated/cohorted patients. However, most of the incidence originated from sporadic sources, which were unaffected by contact isolation. When those sources are elevated, the model predicted that even substantial efforts to interrupt transmission from positive patients would have limited impact on controlling ESBL-E. Conversely, targeting both patient-to-patient transmission and sporadic sources would dramatically diminish incidence.

Several mathematical models have addressed the effect of contact isolation on methicillin-resistant *Staphylococcus aureus* acquisition rates [17,18], but, to our knowledge, not on ESBL-E. We found weak evidence for transmission reduction associated with barrier precautions for isolation measures other than nurse and patient cohorting. While some patient-to-patient transmission was clinically likely (which is why we included it in the model and quantified its magnitude), the statistical signal in our small dataset was not strong enough to be able to rule out the hypothesis that there was no transmission with any degree of certainty. A robust result, however, was that the dominant ESBL-E–acquisition source was not associated with patient-to-patient transmission. These findings are consistent with recent reports indicating that in-hospital ESBL-E transmission is low in the non-outbreak setting [29-31]. Although we referred to non-cross–transmission sources as sporadic, the term “endogenous” has been coined to describe acquisition from a patient’s own flora driven by the selective pressure of antibiotics [21]. Using a comparable model for cephalosporin-resistant Enterobacteriaceae, Bootsma *et al.* found the endogenous route to be the quasi-exclusive source of acquisitions in two intensive care units [20]. Indeed, antibiotic use is recognized as a major risk factor of ESBL-E acquisition, especially in the non-outbreak setting. The mechanism involved might be the disruption of the anaerobic microflora in the intestinal tract, causing the suppression of a defense mechanism (the so-called colonization resistance) against antibiotic-resistant pathogens [32]. The persistently high ESBL-E incidence during our study, despite aggressive detection and isolation, particularly in P_2_, could therefore be explained by unrestricted antibiotic use.

In recent years, ESBL-E have also emerged as important pathogens in the community. Elevated rates were reported and could result in a high influx of colonized patients into hospitals [5,33]. In light of this potentially large reservoir, the usefulness of screening patients at admission, or at all, has been questioned in non-epidemic situations [16,34]. Indeed, our results indicated that screening at admission would have little or no benefit when admission prevalence is low (<5%). For higher levels, they showed that some degree of control could be achieved by screening admitted patients. Admittedly, these results hold only if cross-transmission is extensive, for which we provided only weak evidence in this modeling study but which might be stronger in other settings.

Our study has several limitations. With respect to the mathematical model, a series of assumptions were made that merit being discussed. Patients were assumed to be homogeneous regarding the risk of acquiring or transmitting ESBL-E, even though risk factors for ESBL-E carriage have been described [4]. More generally, like other modeling studies, several factors were omitted that could contribute markedly to ESBL-E spread, particularly staffing ratios and bed occupancy rates [35], even though both quantities were comparable in P_1_ and P_2_ (Table 2). More detailed models, such as individual-based models, are more appropriate to incorporate those factors [36], but at the cost of being more difficult to parameterize when limited data are available.

The assumption that no transmission originated from isolated patients in P_2_ might seem questionable. Adopting another hypothesis, however, would have led to violate the assumption of homogeneous mixing between patients that was made in the model formulation. Admittedly, imperfect separation between patients may have occurred; yet detecting such breaches would have required a close monitoring of staff contacts (e.g. with wearable sensors [37]), and a more elaborate model (e.g. network-based [38]) to integrate those data.

The test for detecting ESBL-E carriage was assumed to have perfect sensitivity. Relaxing that assumption, the sensitivity was estimated at 0.85 (0.56–1), and, therefore, not significantly different from 100%. In addition, assuming an imperfect sensitivity had little impact on the estimates, as judged by a simulation study (Additional file 2).

Concerning the internal validity of our results, because a hygiene-enhancement program was implemented concomitantly, we cannot rule out that the limited impact of patient-to-patient transmission was, indeed, due to that intervention, rather than to isolation measures. Hand-hygiene compliance was estimated from several audits during the study period and was high (>80%). Therefore, cross-transmission can be greater in units where compliance is lower. However, even in such settings, sporadic acquisition would still be expected to be contributory. Molecular-typing methods were not used to verify the findings of our transmission model, a clear limitation of the present study. Nevertheless, the difficulties of considering those methods as the standard reference (e.g. related to the discriminatory power of the method used, the arbitrary definition of an epidemiological linkage between two cases etc.) have been discussed [20,39]. Notwithstanding this point, previous studies reported that, when used, both mathematical modeling and genotyping approaches yielded comparable results [21,40].

Interventions were prompted by an unusually high ESBL-E prevalence in the ward, a situation possibly leading to regression to the mean effects when assessing isolation measures [23]. Finally, our observations, obtained in an endemic setting, cannot be transposed to outbreak settings. Previous reports have shown that ESBL-E outbreaks often involve a particular epidemic and/or virulent clone, and contact isolation was able to eradicate ESBL-E before it became endemic [11,41].

## Conclusions

In conclusion, our results showed that, because of substantial sporadic acquisition sources, contact-isolation measures alone might not suffice to substantially reduce ESBL-E rates in hospital settings. The model could readily be extended to include more features, which might make it applicable to the analysis of other nosocomial pathogens as well. In light of these observations, it would be pertinent to investigate further the interplay between barrier precautions and antibiotic use, and the possible trade-off that might exist to choose the most effective control strategy.

## Competing interests

The authors declare they have no competing interests.

## Authors’ contributions

MDC conceived and coded the model, implemented the statistical analysis, and drafted the manuscript. JRZ conceived of the study design, oversaw data collection, performed the data preparation for modeling and helped draft the manuscript. VA contributed to the data collection and the data cleaning for use in the models. DG contributed content expertise, oversaw the analysis and helped draft the manuscript. All authors read and approved the final manuscript.

## Pre-publication history

The pre-publication history for this paper can be accessed here:

http://www.biomedcentral.com/1471-2334/13/187/prepub

## Supplementary Material

Additional file 1 Supplementary text detailing the model simulation and estimation methods and the results of a sensitivity analysis on fixed parameters. Click here for file

Additional file 2 R code implementing the model and verification of the estimation method on simulated data. Click here for file
