# Supplementary material for: Limits of patient isolation measures to control extended-spectrum beta-lactamase–producing Enterobacteriaceae: model-based analysis of clinical data in a pediatric ward
Source: BMC Infect Dis. 2013 Apr 24;13:187. doi: 10.1186/1471-2334-13-187 (PMC3640926; doi:10.1186/1471-2334-13-187)
Supplement: Additional file 1 — Supplementary text detailing the model simulation and estimation methods and the results of a sensitivity analysis on fixed parameters. [file 1471-2334-13-187-S1.pdf]

Additional file 1

1. Model simulation

The model was simulated using Gillespie's exact algorithm, with 4 state variables (S, C_1, C_2, I) plus an accumulator variable, A, counting the cumulated number of acquisitions over the week and reset at 0 at each new observation. The size of the ward N is kept constant by assuming that each discharge is immediately replaced by an admission into the ward. $\mathbb{I}(.)$ represents an indicator function, such as $\mathbb{I}(I < N_I) = 1$ if $I < N_I$, 0 otherwise.

Table S1 Stochastic events for the isolation model described in the main text

Event	Change	Rate
Discharge/admission	$(S, C_1) \rightarrow (S-1, C_1+1)$	$\mu_S \sigma (1-\epsilon) S$
Acquisition	$(S, C_2) \rightarrow (S-1, C_2+1)$	$(\beta_0 + \beta_1 (C_1 + C_2) + \beta_2 I) S$
Discharge/admission	$(S, I) \rightarrow (S-1, I+1)$	$\mu_S \sigma \epsilon S \times \mathbb{I}(I < N_I)$
Discharge/admission	$(C_1, S) \rightarrow (C_1-1, S+1)$	$\mu_C (1-\sigma) C_1$
Isolation/discharge	$(C_1, I) \rightarrow (C_1-1, I+1)$	$(\delta_1 + \mu_C \sigma \epsilon) C_1 \times \mathbb{I}(I < N_I)$
Discharge/admission	$(C_2, S) \rightarrow (C_2-1, S+1)$	$\mu_C (1-\sigma) C_2$
Discharge/admission	$(C_2, C_1) \rightarrow (C_2-1, C_1+1)$	$\mu_C \sigma (1-\epsilon) C_2 \times \mathbb{I}(I < N_I)$
Isolation/new case detected	$(C_2, I) \rightarrow (C_2-1, I+1)$ $A \rightarrow A+1$	$\delta_2 C_2 \times \mathbb{I}(I < N_I)$
Discharge/ admission	$(C_2, I) \rightarrow (C_2-1, I+1)$	$\mu_C \sigma \epsilon C_2 \times \mathbb{I}(I < N_I)$
Discharge/admission	$(I, S) \rightarrow (I-1, S+1)$	$\mu_I (1-\sigma) I$
Discharge/admission	$(I, C_1) \rightarrow (I-1, C_1+1)$	$\mu_I \sigma (1-\epsilon) I$

2. Model estimation

Model parameters were estimated using the iterated filtering algorithm (MIF). The procedure requires the specification of "algorithmic parameters", which, once convergence has been checked, play no role in the results. For all estimations, we used the same initial variance multiplier $c^2 = 5$ and the same cooling factor $\alpha = 0.95$. Maximum likelihood estimates reported in the main text were computed as the mean of 10 MIF replicates with 20 000 particles and 150 filtering iterations. For likelihood-profile computations, each parameter was fixed at different sampled values and the maximization was performed over the other parameters, as the best of 2 MIF replicates with 5 000 particles and 60 filtering iterations. The profile was then smoothed using a local quadratic regression, and the 95% univariate confidence interval was taken to be $\chi_1^2(0.95)/2 \approx 1.92$ log-likelihood units below the maximum.

3. Sensitivity analyses

In model simulations, two parameters were fixed (isolation rate for acquired cases, δ_2, and percentage of direct isolation in P_2, ϵ) whose exact values were uncertain. Tables S2 and S3 report a sensitivity analysis on those two parameters. As shown, acquisition parameters estimates varied little, *a posteriori* justifying fixing them to any reasonable value within their range of uncertainty.

Table S2 Sensitivity analysis on parameter δ_2. Estimates of acquisition parameters, in day^{-1}, are reported for different values of the time before isolation for acquired cases, $1/\delta_2$. The value used in model simulations and reported in the main text, $1/\delta_2 = 4$ days, is indicated for comparison.

$1/\delta_2$	β_0		β_1		β_2
	P_1	P_2	P_1	P_2	
5 days	0.009	0.006	0.009	0.009	<0.001
3 days	0.008	0.006	0.006	0.005	0.001
4 days	0.009	0.008	0.006	0.006	0.001
2 days	0.008	0.006	0.006	0.007	<0.001

Table S3 Sensitivity analysis on parameter ϵ. Estimates of acquisition parameters, in day^{-1}, are reported for different values of the percentage of direct isolation, ϵ. The value used in model simulations and reported in the main text, $\epsilon = 1$, is indicated for comparison.

ϵ	β_0		β_1		β_2
	P_1	P_2	P_1	P_2	
0.5	0.007	0.005	0.005	0.005	0.001
0.75	0.006	0.006	0.006	0.005	0.001
1	0.009	0.008	0.006	0.006	0.001
