# Supplementary material for: Limits of patient isolation measures to control extended-spectrum beta-lactamase–producing Enterobacteriaceae: model-based analysis of clinical data in a pediatric ward
Source: BMC Infect Dis. 2013 Apr 24;13:187. doi: 10.1186/1471-2334-13-187 (PMC3640926; doi:10.1186/1471-2334-13-187)
Supplement: Additional file 2 — R code implementing the model and verification of the estimation method on simulated data. [file 1471-2334-13-187-S2.zip › ESM2/readme.docx]

**Electronic Supplementary material 2: description of the R code for the isolation model**

The directory ESM2 consists of the following files:

1. isolmodel.c: contains the model equations in native code
2. create_isolmodel.R: R script creating the pomp model
3. profile_loglike: function to profile over one parameter
4. main.R: simulate and plot 100 weeks of data; performs inference on these simulated data
5. profiles_perfect_sensitivity.pdf, profiles_imperfect_sensitivity.pdf: results of estimations when the screening test is assumed perfect () or imperfect ()

Model parameters were those reported for P1 in the main text (Table 1). The same set of parameters was also estimated (Table S4). For each parameter, the log-likelihood profile was computed by fixing to several values in a prespecified range (Table S4) and maximizing the log-likelihood over all parameters, excluding . For the results represented in the pdf files, 50 points were tested per interval, with 2 estimation procedures per point, for a total of 100 estimation procedures for each parameter. All estimations used the same algorithmic parameters for the iterated filtering algorithm (Table S5); those were the same than reported in the electronic supplementary material 1, except for the number of particles set to 2,000. Each parameter profiling procedure took ~20 hours on a desktop computer.

On the pdf files, grey points represent the profile log likelihood values, grey lines the smoothed profiles (obtained by a local quadratic regression, span 0.75); vertical black dashed lines encompass the 95% confidence interval while the vertical continuous black and red lines are the estimated value and the true value, respectively.

| **Parameter** | **Unit** | **Fixed value** | **Range tested** |
| --- | --- | --- | --- |
|  | day | 0.01 | (0–0.03) |
|  | day | 0.005 | (0–0.03) |
|  | day | 0.001 | (0–0.01) |
|  | — | 0.2 | (0.1–0.3) |

Table S4: fixed values for the estimated parameters, and range of values tested for the log-likelihood profiles.

| **Parameter** | **Description** | **Value** |
| --- | --- | --- |
|  | Number of particles |  |
|  | Number of filtering iterations | 60 |
|  | Cooling factor | 0.95 |
|  | Initial variance multiplier | 5 |

Table S5: Algorithmic parameters used for the results presented in the pdf files.
