# Supplementary figures and images for: Limits of patient isolation measures to control extended-spectrum beta-lactamase–producing Enterobacteriaceae: model-based analysis of clinical data in a pediatric ward
Source: BMC Infect Dis. 2013 Apr 24;13:187. doi: 10.1186/1471-2334-13-187 (PMC3640926; doi:10.1186/1471-2334-13-187)

**b0**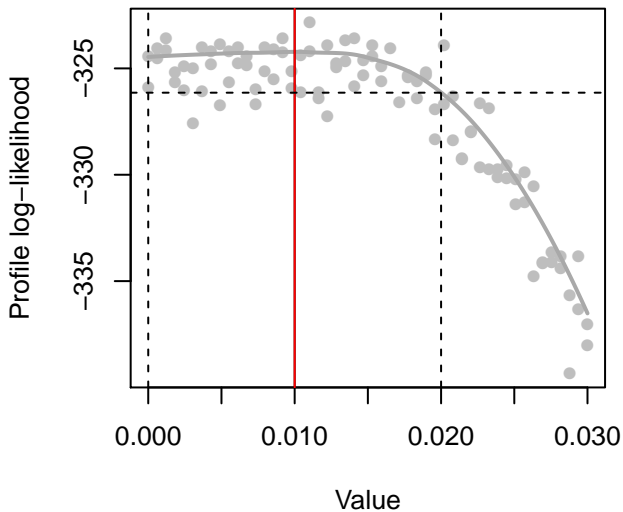**b1**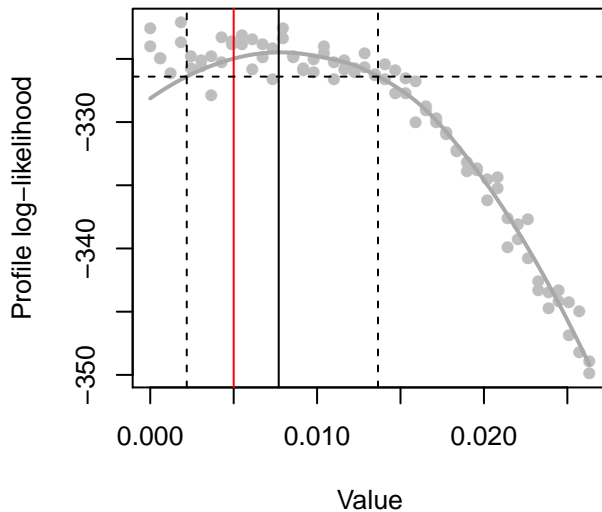**b2**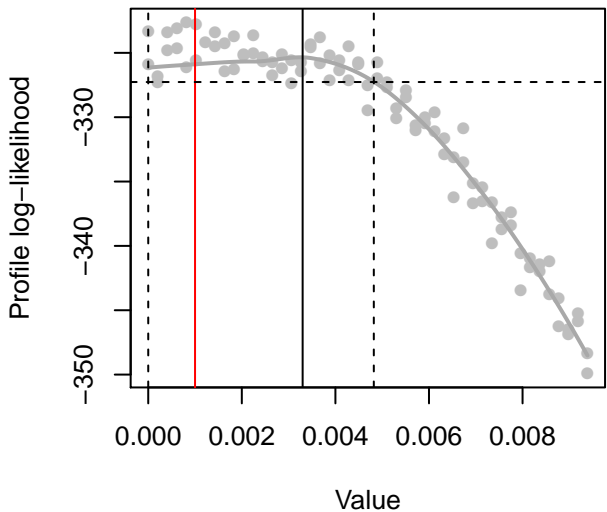**sigma**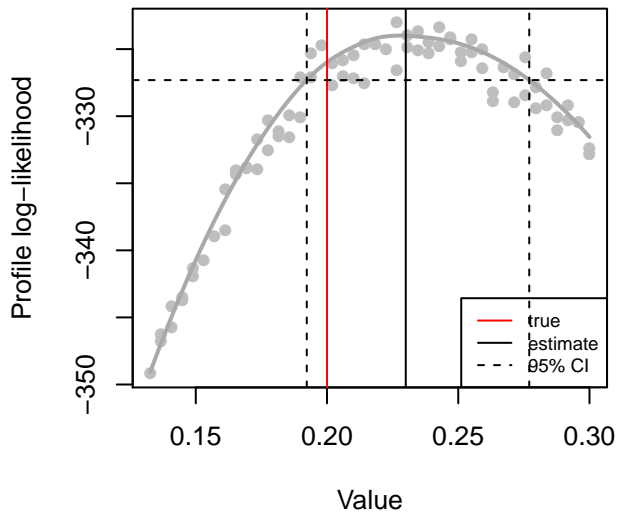

Supplement: Additional file 2 — R code implementing the model and verification of the estimation method on simulated data. [file 1471-2334-13-187-S2.zip › ESM2/profiles_imperfect_sensitivity.pdf]

**b0**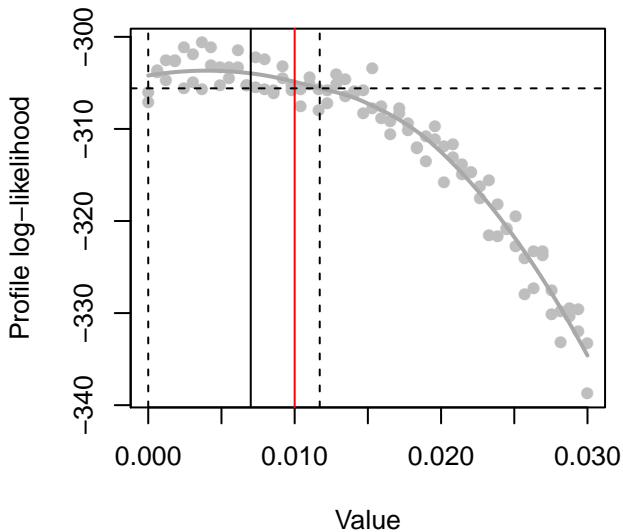**b1**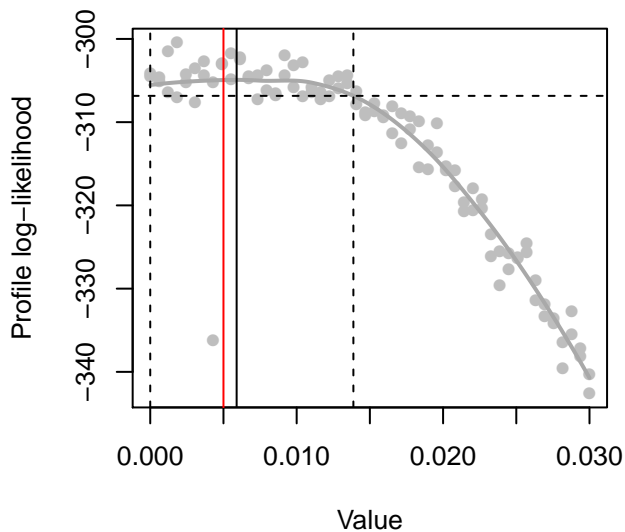**b2**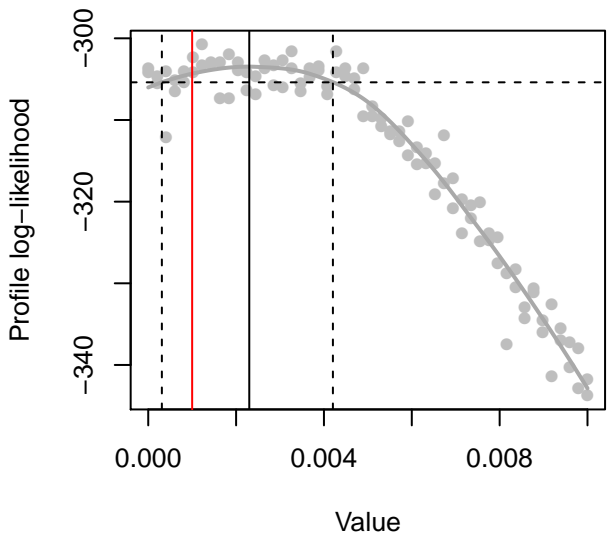**sigma**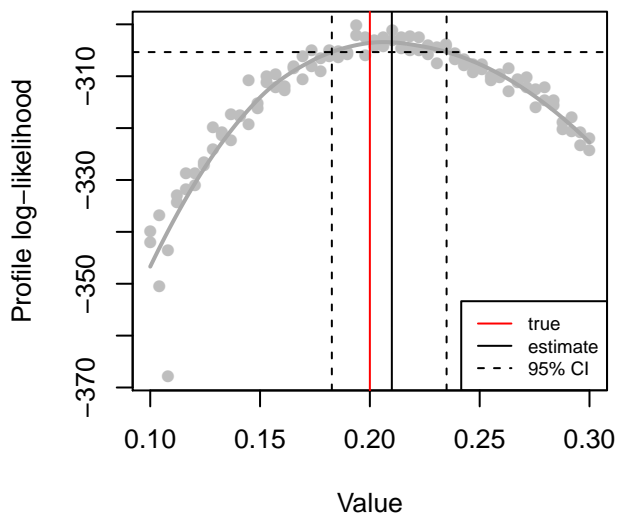

Supplement: Additional file 2 — R code implementing the model and verification of the estimation method on simulated data. [file 1471-2334-13-187-S2.zip › ESM2/profiles_perfect_sensitivity.pdf]
